# Cellular cholesterol loss by DHCR24 knockdown leads to Aβ production by changing APP intracellular localization

**DOI:** 10.1016/j.jlr.2023.100367

**Published:** 2023-04-01

**Authors:** Yue Huang, Wenbin Zhang, Xiaorou Guo, Ying Zhang, Junfeng Wu, Hengbing Zu

**Affiliations:** Department of Neurology, Jinshan Hospital Affiliated to Fudan University, Shanghai, China

**Keywords:** Alzheimer’s disease, APP, Amyloid-β, DHCR24, Cholesterol, Pathogenesis

## Abstract

For the past 20 years, the majority of cell culture studies reported that increasing cholesterol level increases amyloid-β (Aβ) production. Conversely, other studies and genetic evidences support that cellular cholesterol loss leads to Aβ generation. As a highly controversial issue in Alzheimer’s disease pathogenesis, the apparent contradiction prompted us to again explore the role of cellular cholesterol in Aβ production. Here, we adopted new neuronal and astrocytic cell models induced by 3β-hydroxysterol-Δ24 reductase (DHCR24), which obviously differ from the widely used cell models with overexpressing amyloid precursor protein (APP) in the majority of previous studies. In neuronal and astrocytic cell model, we found that deficiency of cellular cholesterol by DHCR24 knockdown obviously increased intracellular and extracellular Aβ generation. Importantly, in cell models with overexpressing APP, we found that APP overexpression could disrupt cellular cholesterol homeostasis and affect function of cells, coupled with the increase of APP β-cleavage product, 99-residue transmembrane C-terminal domain. Therefore, we suppose the results derived from the APP knockin models will need to be re-evaluated. One rational explanation for the discrepancy between our outcomes and the previous studies could be attributed to the two different cell models. Mechanistically, we showed that cellular cholesterol loss obviously altered APP intracellular localization by affecting cholesterol-related trafficking protein of APP. Therefore, our outcomes strongly support cellular cholesterol loss by DHCR24 knockdown leads to Aβ production.

A tight link between Alzheimer’s disease (AD) pathology and lipids was already observed more than a century ago by Alois Alzheimer, who described a higher occurrence of “lipoid granules” in postmortem AD-brain tissue as a third pathological hallmark of the disease (1, 2). Moreover, accumulating biological, epidemiological, and genetic studies showed that cholesterol loss prevalently involved in AD pathogenesis, including tau pathology, apoptosis, and synaptic injuries, and inhibition of autophagy (3, 4, 5). However, in terms of amyloid-β (Aβ) generation, a well-recognized pathological feature in AD, the vast majority of the cell culture studies have reported that elevated cellular cholesterol level increases Aβ production (6, 7, 8, 9). Only a few evidences suggested that decreased cholesterol level promotes Aβ generation (10, 11). Given that enormous evidence of other AD pathologies supported that cholesterol shortage may contribute to the development of AD (3, 4, 5), the divergent view of cholesterol in Aβ encourages a reconsideration of how cholesterol affects Aβ metabolism.

Intriguingly, a series of evidence demonstrated that in the brain of hereditary AD animals and patients, there are susceptible genes and gene polymorphism in the molecules responsible for the transport, uptake, and intracellular trafficking of cholesterol, including apolipoprotein E, ATP binding cassette transporters, low density lipoprotein receptor family, and Niemann-Pick Type C 1/2 (NPC1 and NPC2). Furthermore, these genic alterations easily lead to the decrease of cholesterol transport or uptake and blockade of intracellular cholesterol trafficking, resulting in cellular cholesterol deficiency/loss (12, 13, 14, 15, 16). What’s more, genetic data strongly suggest that cellular cholesterol deficiency could contribute to AD pathology, such as Aβ production and deposit, tau protein accumulation, and synaptic impairment (3, 4, 5, 10, 11). In view of apparent contradictory findings of previous researches, the role of cholesterol in processing of amyloid precursor protein (APP) comes under close scrutiny: does high or low cellular cholesterol level contribute to Aβ generation (17, 18)? Therefore, as a highly controversial issue in AD pathogenesis, we have to again explore the role of cellular cholesterol in Aβ production.

In addition, the source of Aβ deposition remains a controversial issue in AD brain (19, 20, 21). Generally, neurons have been widely recognized as the primarily sources of Aβ to date; however, similar to neurons, astrocytes also express APP, the substrate, and related lyases, indicating astrocytes equip with the machine to produce Aβ. What’s more, intracellular deposits of Aβ were found in activated astrocytes surrounding Aβ plaques (22, 23). Moreover, taken account of the fact that astrocytes are the main sources of cholesterol in brain, the role of astrocytes in the production of Aβ should be received more attention, which may help us to further understand the role of astrocytes in AD pathogenesis. Therefore, it is necessary to detect whether altered cellular cholesterol level can influence APP cleavage in astrocytes.

Most notably, supporting the view that high cellular cholesterol increases amyloid beta production, nearly completely is derived from the cell or animal models carrying a mutant human APP (6, 7, 8, 9). Because APP, Aβ, and other APP fragments might be very important inhibitory regulators that exert effect on cholesterol synthesis, overexpressing APP and other APP fragments could inhibit the de novo cholesterol synthesis and result in the disruption of cellular cholesterol homeostasis, which could affect the structure and function of cell models and outcomes of the previous studies (24, 25, 26). In cellular cholesterol synthesis and homeostasis, 3β-hydroxysterol-Δ24 reductase (DHCR24) is known as a heavily key synthetase (17, 27). In our previous study, we found cellular cholesterol levels could be effectively modulated by DHCR24 knockin or knockdown in neuronal and astrocytic cell lines. Thus, in the present study, we adopt new neuronal and astrocytic cell model of cellular cholesterol deficiency by DHCR24 knockdown and increased cellular cholesterol level by DHCR24 knockin, which could closely resemble physiological and pathological situations (28, 29, 30). Here, we could present an alternative and more suitable cell model. To further elucidate the relationship between cellular cholesterol level and Aβ production, we investigate the effect of changes in cellular cholesterol content mediated by DHCR24 on Aβ production in neurons and astrocytes and further compared the difference of Aβ generation between neurons and astrocytes.

## MATERIALS AND METHODS

### Antibodies

A rabbit antibody against C terminus of APP (Y188, Abcam, ab32136) was used to detect APP-C83 and APP-C99; a mouse antibody against amino acids 66–81 of the N terminus of APP (22C11, Millipore, MAB348) was used to detect full-length APP.

Rabbit anti-BACE-1(ab10716), anti-Rab5(ab218624), anti-LAMP1(ab20894), and anti-Flotillin1(ab133497) were purchased from Abcam (British). Rabbit anti-DHCR24 (#2033), anti-SorLA (#79322), and mouse anti-GAPDH (#2118) were obtained from Cell Signaling Tech.

### Cell culture and lentivirus-mediated transfection

Mouse hippocampal neuronal cell line (HT22) was obtained from Fu Heng (Cat# FH1027), and Astrocyte Type I clone cell line (C8D1a) was obtained from ZQXZbio (Cat# ZQ0566) and were grown in 4.5 g/L glucose Dulbecco’s Modified Eagle Medium (Gibco, China), supplemented with 10% fetal bovine serum (Gibco). In addition, cells were cultured at 37°C with 5% CO2 according to the manufacturer’s instructions.

Cells were transfected by lentivirus-mediated DHCR24 cDNA and DHCR24-shRNA or empty vectors, respectively (Jima, Shanghai). Firstly, these cells were transfected with lentivirus for 24 h when cells density was about 50%. Afterwards, we continued to cultivate the cells for 48 h in fresh medium. Subsequently, to screen out cells which had successfully transfected lentivirus, we cultivated cells in fresh medium supplemented with an appropriate concentration of puromycin (2 μg/ml). The shRNA sequence of DHCR24 is as follow: (5′-GCGAGGAATTCTGGGAGATGTTCGA-3′). Additionally, the method for APP overexpressing cell model construction was the same as above.

### Real-time PCR analysis

Total RNA was extracted from cells using RNAiso Plus Kits (Takara, Japan) according to protocol recommended. Reverse transcription of mRNA was performed by PrimeScript™ RT Master Mix kits (Takara). Gene expression was quantitatively measured using TB Green Premix Ex Taq II kits (Takara) with specific primers (Table 1). All experiments were performed at least three times.

**Table 1:** Primer sequences of genes associated with cholesterol biosynthesis and APP cleavage

Genes		Primer Sequences
SQLE	Forward	AGTTCGCTGCCTTCTCGGATA
	Reverse	GCTCCTGTTAATGTCGTTTCTGA
HMGCR	Forward	ATGCCTTGTGATTGGAGTTG
	Reverse	GTTACGGGGTTTGGTTTATT
SREBP2	Forward	GCAGCAACGGGACCATTCT
	Reverse	CCCCATGACTAAGTCCTTCAACT
DHCR24	Forward	CGCTGCGAGTCGGAAAGTA
	Reverse	GTCACCTGACCCATAGACACC
DHCR7	Forward	AGGCTGGATCTCAAGGACAAT
	Reverse	GCCAGACTAGCATGGCCTG
APP	Forward	TGAATGTGCAGAATGGAAAGTG
	Reverse	AACTAGGCAACGGTAAGGAATC

APP, amyloid precursor protein; DHCR7, 7-dehydrocholesterol reductase; DHCR24, 3β-hydroxysterol-Δ24 reductase; HMGCR, hydroxymethyl glutaryl-CoA reductase; SREBP2, cholesterol regulatory element binding protein 2; SQLE, squalene epoxidase.

### Filipin III staining

Filipin staining is widely used as a method to measure unesterified cholesterol levels (31). Firstly, cells were seeded in coverslips and were cultured for 12–18 h. After washed by phosphate-buffered saline (PBS) three times, the cells were fixed via 4% paraformaldehyde for 20 min at room temperature. Afterwards, cells were treated with filipin III (0.1 mg/ml, Absin, China) for 30 min at room temperature. Besides, cells also staining with propidium iodide (PI, 0.35 μg /ml) for 5 min. Finally, filipin and PI staining fluorescence intensity were measured using a confocal laser scanning microscope (Leica sp5, Germany) at 405 nm.

### Free cholesterol analysis by cholesterol enzyme link assay

First, cells were seeded in plates and cultivated for 12–18 h. In the next day, all plates were washed three times by PBS, and then cells were collected into Eppendorf tubes separately and immersed in appropriate amount of lysis solution in 10 min. Only the supernatants were collected and measured by a free cholesterol quantitative kit (Applygen, Beijing, # E1016) according to protocol recommended by manufacturer. The concentration of protein in supernatants also was measured by BCA kits (Merk, Shanghai) to normalized free cholesterol content.

### Western blotting analysis

All proteins are extracted from cells using SDS lysis buffer (Merk) supplemented with both phosphatase inhibitor (1:100 Millipore, Shanghai) and protease inhibitor (1:100 Millipore). After removing cell debris by centrifugation at 12,000 × rpm, the concentration of protein was measured by BCA assay kits (Millipore). Subsequently, an equal amount (20 ug) of proteins was loaded on 10% polyacrylamide gels (Cat# PG112, EpiZyme Biotechnology, China) according to their concentrations. Afterward, proteins were transferred onto polyvinylidene fluoride membranes, and the membranes were blocked with 5% skimmed milk at room temperature for 1 h. Besides, the membranes were incubated with primary antibodies at 4°C overnight, such as anti-BACE-1(1:1000), anti-Flotillin-1 (1:5000), anti-APP (1:1000), anti-SorLA (1:1000), anti-CTF (1:1000), anti-DHCR24 (1:1000), and anti-GAPDH (1:10,000). In the next day, the membranes were incubated with horseradish peroxidase–conjugated secondary antibodies (1:5,000) for 1 h at room temperature, and the immunoreactive bands were detected by Immobilon ECL Ultra Western HRP (Millipore, Shanghai). All results had been repeated at least three times.

### The measurement of Aβ by ELISA kits

To detect the amount of secreted Aβ40 and Aβ42 in neuronal and astrocytic cell lines, we seeded an equal number of cells in vessels and cultured cells for 12 h. After checking that the cell density of each group is roughly 50%, we removed culture medium completely and gently washed cells by PBS three times. Afterwards, 4 ml fresh culture medium was added to each culture flask, and then, we cultured cells for another 48 h. Subsequently, the mediums were collected and were centrifugated at 1000 rpm for 5 min to remove cell debris. Ultimately, the supernatants were measured by specific ELISA kits (IBL, Germany, # 27720 and # 27721) according to protocol recommended by manufacturer.

Additionally, to detect the amount of intracellular Aβ40 and Aβ42, firstly we extracted proteins from cells and measured the concentration of protein extraction via BCA kits. Subsequently, all samples were measured by ELISA kits according to protocol recommended by manufacturer. Most notably, the amount of intracellular Aβ40 and Aβ42 should were normalized by total protein concentration, which was measured by BCA kits. These experiments duplicated at least three times, and the data were acquired from independent experiments.

### Immunostaining analysis

Firstly, cells were seeded in coverslips and cultivated for 12–18 h. And then, cells were washed three times by PBS before being fixed via 4% paraformaldehyde for 20 min and exposed to 0.5% Triton X-100 for 30 min at room temperature. Afterward, cells were incubated with anti-APP antibody (1:500) in combination of specific marker proteins antibodies, including anti-Rab5 (1:100), anti-TGN46 (1:100), and anti-LAMP1 (1:100) overnight. After another wash, cells were incubated with both Alexa Fluor 488 and 594-conjugated secondary antibody (1:1000) for an hour at room temperature. After being washed, the cells were exposed with DAPI (Beyotime, Shanghai) about 5 min and were measured by confocal laser scanning microscope (Leica sp5, Germany).

Images were obtained using ×63 and ×100 oil immersion objectives; the colocalization of APP with specific marker proteins was quantified using Coloc2 plugin of ImageJ and presented using Mander’s correlation coefficient. To analyzed the size distribution of early endosomes puncta, we firstly converted picture unite from pixels into microns via Spatial calibration plugin of Image Pro Plus and counted the numbers of early endosomes within limited sizes and the areas of early endosomes, respectively, using Count/Size plugin of Image-Pro Plus software. These experiments duplicated at least three times, and the data were acquired from independent experiments.

### Statistical analysis

All values were presented as mean ± standard deviation (mean ± SD) of at least three experiments, and analysis of variance was performed using GraphPad Prism 8 (GraphPad, CA). To compare statistical differences among each group, one-way ANOVA was used. The definition of statistical no significance (ns) is *P* > 0.05; In addition, ∗*P* < 0.05, ∗∗*P* < 0.01; ∗∗∗*P* < 0.001; and ∗∗∗∗*P* < 0.0001 were all considered statistically significant.

## RESULTS

### Cellular cholesterol level was effectively regulated by DHCR24 knockdown or knockin in both astrocytic C8D1A and neuronal HT22 cell lines

**Fig. 1:**
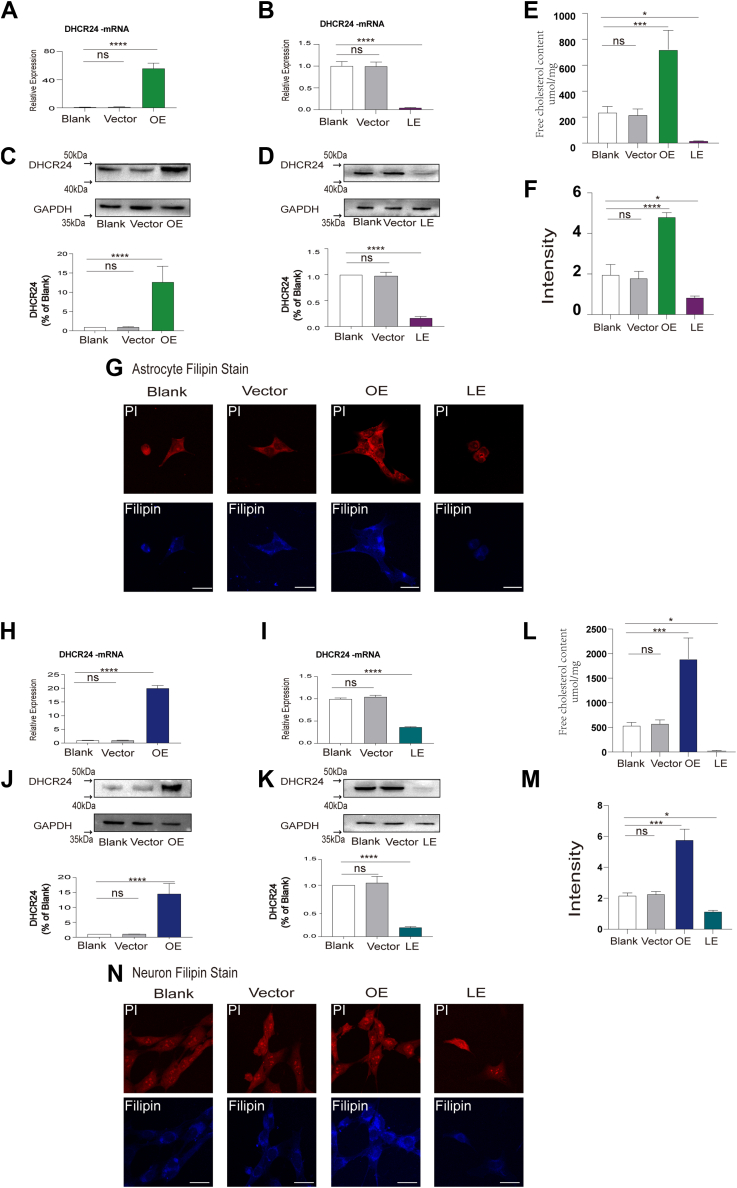
Cellular cholesterol level was effectively regulated by DHCR24 knockdown or knockin in both neuronal and astrocytic cell lines. A, B: Quantification of DHCR24 mRNA expression in blank, vector, overexpressing (OE), and lower-expressing (LE) DHCR24 astrocytic cell lines. C, D: Representative western blots of DHCR24 and quantification of relative DHCR24/GAPDH ratio of immunoblots from cell lysates from in blank, vector, OE, and LE DHCR24 astrocytic cell lines. Value for blank astrocytes were set to 1. E: Quantification of free cholesterol content in blank, vector, OE, and LE DHCR24 astrocytic cell lines. F: Quantification of Filipin stain immunofluorescence mean intensity in blank, vector, OE, and LE DHCR24 astrocytic cell lines. G: Images of blank, vector, OE, and LE DHCR24 astrocytic cell lines, which were stained by Filipin (blue) and PI (red). Scale bars = 20um. H–I: Quantification of DHCR24 mRNA expression in blank, vector, OE, and LE DHCR24 neuronal cell lines. J–K: Representative western blots of DHCR24 and quantification of relative DHCR24/GAPDH ratio of immunoblots from cell lysates from in blank, vector, OE, and LE DHCR24 neuronal cell lines. Value for blank neurons were set to 1. L: Quantification of free cholesterol content in blank, vector, OE, and LE DHCR24 neuronal cell lines. M: Quantification of Filipin stain immunofluorescence mean intensity in blank, vector, OE, and LE DHCR24 neuronal cell lines. N: Images of in blank, vector, OE, and LE DHCR24 neuronal cell lines, which were stained by Filipin (blue) and PI (red). Scale bars = 20um. Statistics were analyzed using one-way ANOVA followed by Turkey’ posttest; n = 3 independent experiments. Mean ±SD. ∗ *P* < 0.05, ∗∗*P* < 0.01, ∗∗∗*P* < 0.001, ∗∗∗∗*P* < 0.0001.

To detect the effect of cholesterol mediated by DHCR24 on Aβ production, we knockin or knockdown DHCR24 to construct cell models with different cholesterol levels via lentiviral vectors in neuronal and astrocytic cell lines. Compared to blank and vector cells, expression level of DHCR24 gene and protein were significantly decreased in lower expressing DHCR24 cells, whereas were significantly elevated in overexpressing DHCR24 cells (Fig. 1A–D, H–K). Moreover, to further confirm the critical role of DHCR24 in cholesterol biosynthesis, we measured cellular free cholesterol level using both cholesterol enzyme link assays and Filipin III staining (31). Herein, we observed the mean fluorescence signal intensity of filipin staining, and free cholesterol content were markedly declined in neuronal and astrocytic cell lines with lower-expressing DHCR24, whereas increased in overexpressing DHCR24 cells (Fig. 1E–G, L–N). Apart from total free cholesterol, in our previous studies, we found that DHCR24 downregulation inhibited intracellular cholesterol esterification by decreased ACAT gene expression. Moreover, the results of Oil Red O staining, a fat-soluble dye that specifically stains triglycerides and cholesteryl oleate but no other lipids, further supported a reduction of intracellular cholesterol ester content in DHCR24 lower expression cells compared to control group (30). These data above strongly supported that DHCR24 acts as an extraordinary regulator in total cellular cholesterol level, and it can be an appropriate tool to constitute experimental models with different cellular cholesterol levels in AD research.

### Cellular cholesterol deficiency mediated by DHCR24 knockdown increased APP cleavage and booted Aβ production in both astrocytic and neuronal cell lines

**Fig. 2:**
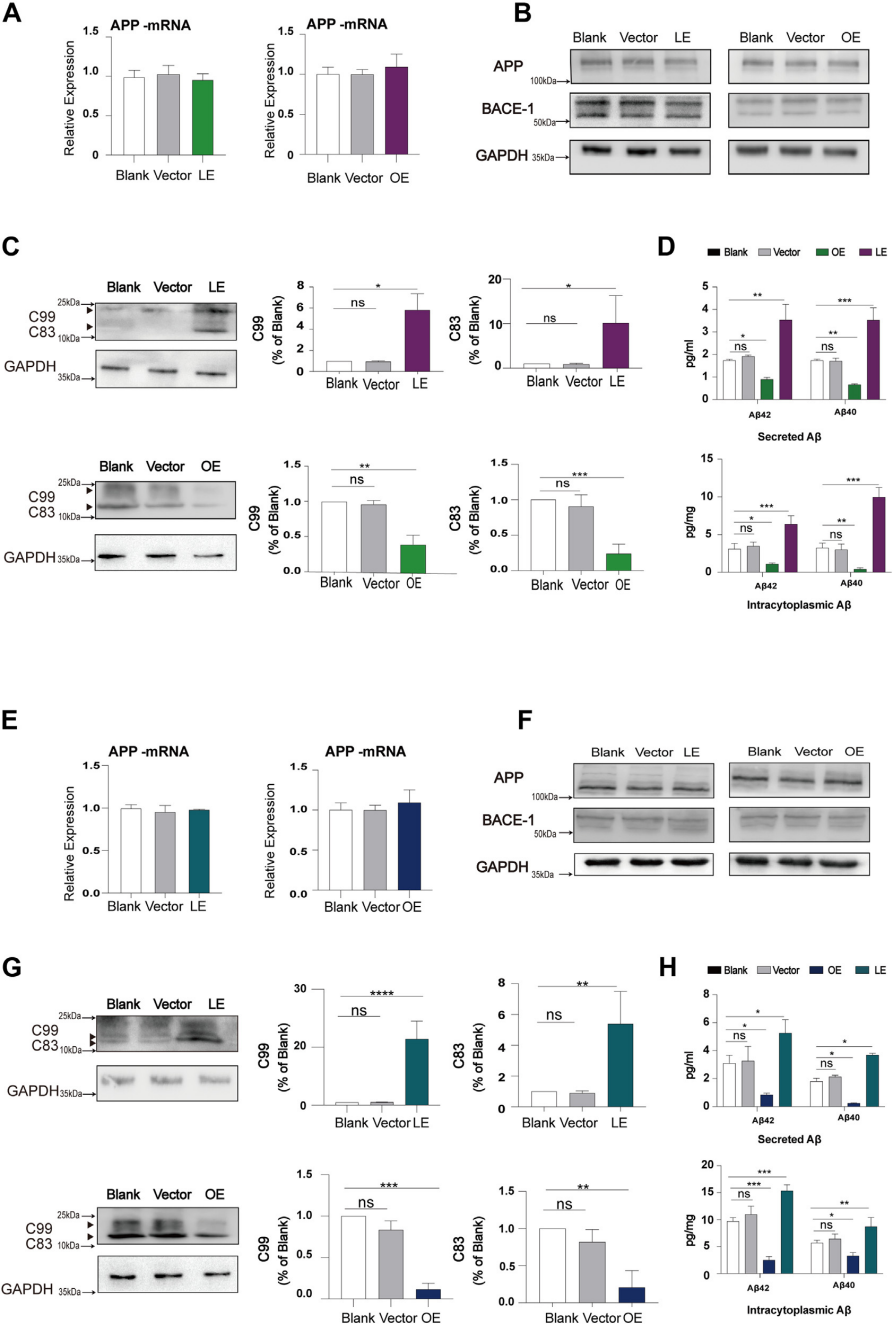
Cellular cholesterol deficiency mediated by DHCR24 knockdown increased APP cleavage and booted Aβ production in both astrocytic and neuronal cell lines. A: Quantification of APP mRNA expression in blank, vector, overexpressing (OE), and lower-expressing (LE) DHCR24 astrocytic cell lines. B: Representative western blots of APP (using 22C11, an antibody against N terminus of APP), BACE-1 from cell lysates from blank, vector, OE, and LE DHCR24 astrocytic cell lines. Value for blank astrocytes were set to 1. C: Representative western blots of C99 and C83 (using Y188, an antibody against C terminus of APP) and quantification of relative C99 /GAPDH ratio and relative C83/GAPDH ratio of immunoblots from cell lysates from blank, vector, OE, and LE DHCR24 astrocytic cell lines. Value for blank astrocytes were set to 1. D: Intracellular and secreted Aβ42 and Aβ40 in blank, vector, OE, and LE DHCR24 astrocytic cell lines, measured by ELISA. The amount of intracellular Aβ42 and Aβ40 were normalized according to the protein concentration of cell lysates. E: Quantification of APP mRNA expression in blank, vector, OE, and LE DHCR24 neuronal cell lines. F: Representative western blots of APP (using 22C11, an antibody against N terminus of APP), BACE-1 from cell lysates from blank, vector, OE, and LE DHCR24 neuronal cell lines. Value for blank astrocytes were set to 1. G: Representative western blots of C99 and C83 (using Y188, an antibody against C terminus of APP) and quantification of relative C99 /GAPDH ratio and C83/GAPDH ratio of immunoblots from cell lysates from blank, vector, OE, and LE DHCR24 neuronal cell lines. Value for blank astrocytes were set to 1. H: Intracellular and secreted Aβ42 and Aβ40 in blank, vector, OE, and LE DHCR24 neuronal cell lines, measured by ELISA. The amount of intracellular Aβ42 and Aβ40 were normalized according to the protein concentration of cell lysates. Statistics were analyzed using one-way ANOVA followed by Turkey’ posttest; n = 3 independent experiments. Mean ± SD. ∗ *P* < 0.05, ∗∗*P* < 0.01, ∗∗∗*P* < 0.001, ∗∗∗∗*P* < 0.0001.

Although DHCR24 could not influence the expression of APP and BACE-1 (Fig. 2A, B, E, F), we found that cellular cholesterol deficiency could prompt APP processing; thus, the amount of α/β-CTF (C83 and C99) was increased in neuronal and astrocytic cell lines with lower-expressing DHCR24, compared to blank and vector cells (Fig. 2C, G). Importantly, in concert with increased APP β-cleavage, we found cholesterol loss could increase production of intracellular/extracellular (secreted) Aβ40 and Aβ42 (Fig. 2D, H). On the contrary, in neuronal and astrocytic cell lines with overexpressing DHCR24, we found that increased cellular cholesterol reduced α/β-CTF (C83 and C99) generation, as well as intracellular and secreted Aβ40 and Aβ42 production (Fig. 2C, D, G, H). Altogether, our findings suggested that cellular cholesterol deficiency mediated by DHCR24 knockdown increased APP processing and Aβ40/42 generation.

### Overexpression of APP disrupted cellular cholesterol homeostasis and led to increase of APP β-cleavage in both neuronal and astrocytic cell lines

**Fig. 3:**
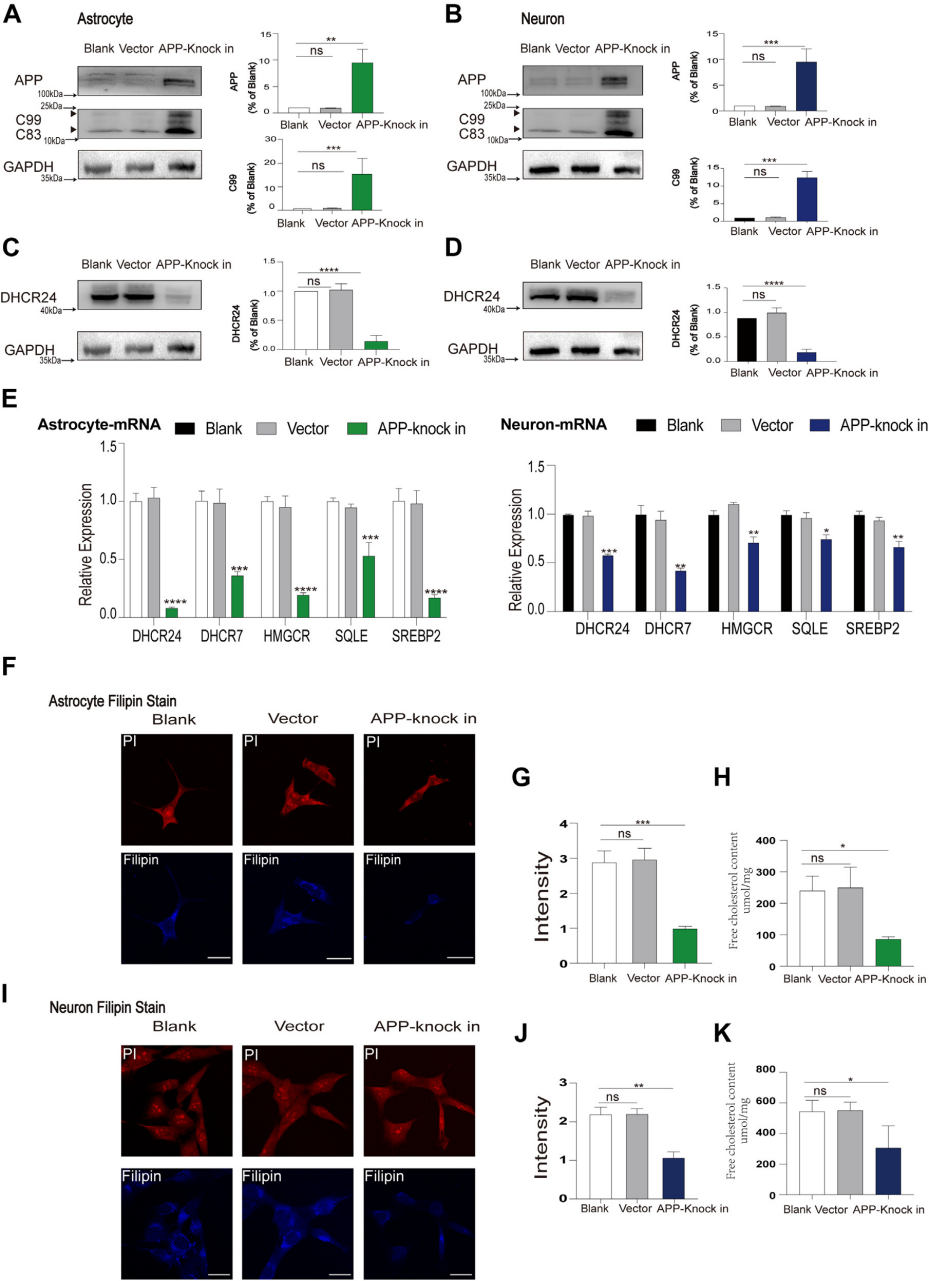
Overexpression of APP disrupted cellular cholesterol homeostasis and led to increase of APP β-cleavage in both neuronal and astrocytic cell lines. A, B: Representative western blots of APP and C99 and quantification of relative APP/GAPDH ratio of immunoblots from cell lysates from blank, vector astrocytes/ neurons, and astrocytes/neurons transfected with APP cDNA. Value for blank astrocytes were set to 1. C, D: Representative western blots of DHCR24 and quantification of relative DHCR24/GAPDH ratio of immunoblots from cell lysates from blank, vector astrocytes/neurons, and astrocytes/neurons transfected with APP cDNA. Value for blank astrocytes were set to 1. Note: images of immunoblots for APP, CTFs, DHCR24, and GAPDH were cropped from the same membrane, thus (A, C), as well as (B, D) shared the same GAPDH immunoblot image. E: Overexpression of APP inhibited several cholesterol-associated synthetases expression in astrocytes/neurons as determined by mRNA expression. F, I: Images of blank, vector astrocytes/neurons, and astrocytes/neurons transfected with APP cDNA, which were stained by Filipin (blue) and PI (red). G, J, Quantification of Filipin stain immunofluorescence mean intensity in blank, vector astrocytes/neurons, and astrocytes/neurons transfected with APP cDNA. Scale bars = 20um. H, K: Quantification of free cholesterol content in blank, vector astrocytes/neurons, and astrocytes/neurons transfected with APP cDNA. Statistics were analyzed using one-way ANOVA followed by Turkey’ posttest; n = 3 independent experiments. Mean ± SD. ∗ *P* < 0.05, ∗∗*P* < 0.01, ∗∗∗*P* < 0.001, ∗∗∗∗*P* < 0.0001.

Based on the discrepancy between our outcomes and the previous results derived from the APP knockin models, we hypothesize that the different results may attribute to the two kinds of different cell models and APP’s effect on cellular cholesterol homeostasis. Because several studies found that APP and its fragments affect cellular cholesterol biosynthesis (24, 25, 26). Notably, in our experiment, we found that elevated APP protein expression promoted the significant increase of APP β-cleavage product, 99-residue transmembrane C-terminal domain (C99, also known as β-CTF), indicating the increase of Aβ production in both neuronal and astrocytic cell lines (Fig. 3A, B). Meantime, overexpression of APP significantly decreased DHCR24 protein expression, suggesting inhibition of cellular cholesterol de novo synthesis in both neuronal and astrocytic cell lines (Fig. 3C, D). Above results are coincided with previous study, supporting elevated APP and its cleavage products could inhibit cholesterol synthesis (32, 33). Collectively, our outcomes indicated that APP overexpression could lead to increase of APP β-cleavage and Aβ generation.

Additionally, the mRNA expressions of major cholesterol synthetases, such as cholesterol regulatory element binding protein 2 (SREBP2), hydroxymethyl glutaryl-CoA reductase (HMGCR), squalene epoxidase (SQLE), 7-dehydrocholesterol reductase (DHCR7), and DHCR24, were significantly decreased in APP overexpressing neuronal and astrocytic cell lines (Fig. 3E). Furthermore, the results from filipin staining and cholesterol enzyme link assays evidenced a decrease of cellular cholesterol level in APP knockin neuronal and astrocytic cell lines (Fig. 3F–K). Thus, our data further revealed that APP overexpression can markedly inhibit cellular cholesterol biosynthesis, resulting in a cellular cholesterol deficiency/loss.

### Cellular cholesterol deficiency could lead to impairment of early endosome’s function and altered localization of APP in trans Golgi network

Intriguingly, APP trafficking in endosomal recycling pathway has always been a focus in the research about the regulation of cellular cholesterol on Aβ generation (34, 35). To reveal mechanism underlying the effect of cholesterol on Aβ generation, we concentrated on investigating the alteration of APP localization in endosomal recycling pathway. Physiologically, it has been reported that majority of APP in early endosomes were subsequently sorted to trans Golgi network (TGN) and other intracellular compartments (36). To determine full-length of APP’s intracellular localization, we labeled APP utilized an antibody, called 22C11, which specifically targets N-terminus fragment of APP to exclude unwanted disturbing signal from CTFs (37, 38).

**Fig. 4:**
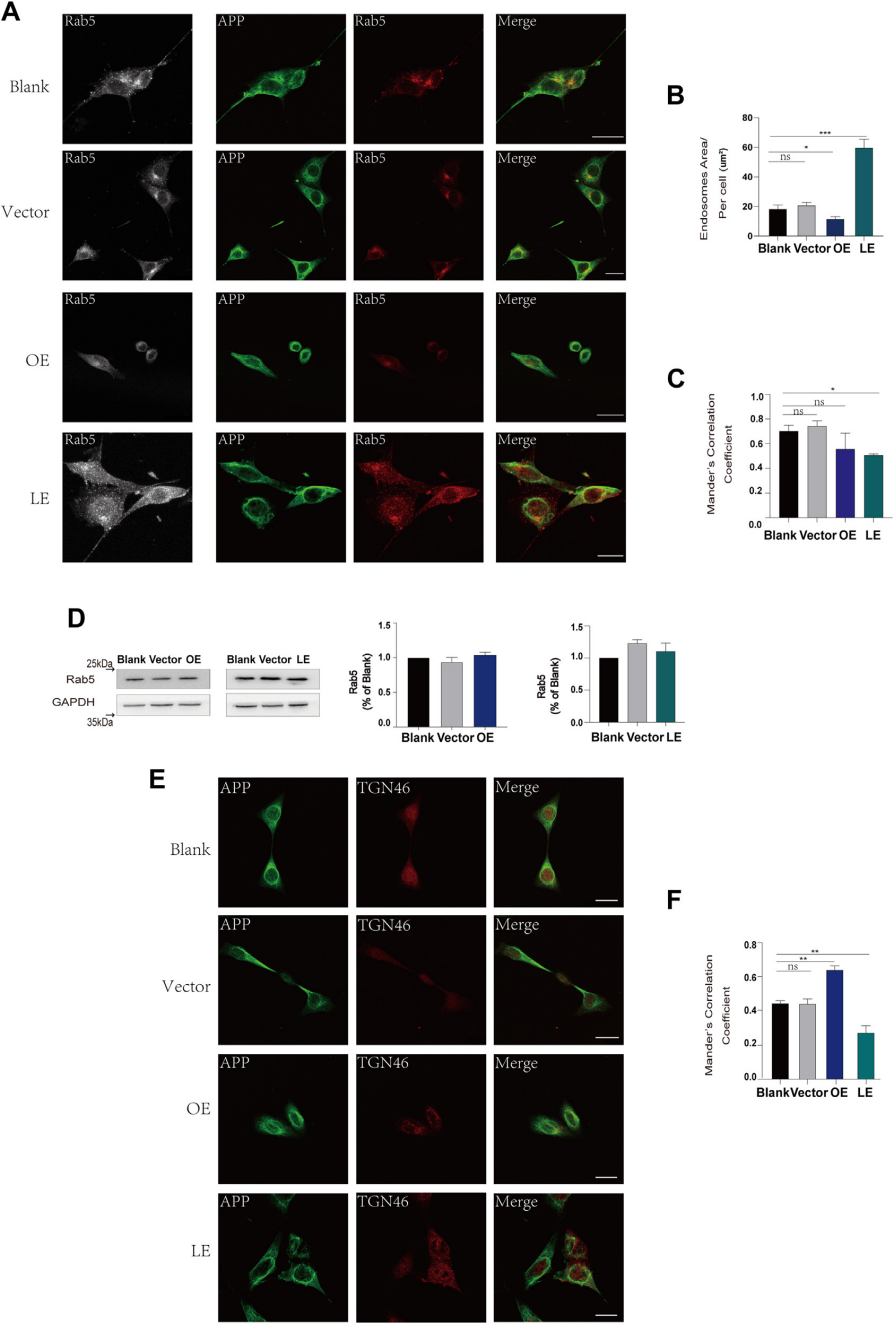
Cellular cholesterol deficiency leaded to impairment of early endosome function and altered localization of APP in TGN. A: Representative immunofluorescence images of blank, vector, overexpressing (OE), and lower-expressing (LE) DHCR24 neuronal cell lines, and these cells were stained with APP (green) and Rab5 (gray and red). Scale bars = 20um. B: Mean Rab5 puncta area per cell in blank, vector, OE, and LE DHCR24 neuronal cell lines. n = 5 images each group. C: Quantification of colocalization of Rab5 with APP measured by ImageJ software. D: Representative western blots of Rab5 and quantification of relative Rab5/GAPDH ratio of immunoblots from cell lysates from blank, vector, OE, and LE DHCR24 neuronal cell lines. E: Representative immunofluorescence images of blank, vector, OE, and LE DHCR24 neuronal cell lines, and these cells were stained with APP (green) and TGN46 (red). Scale bars = 20um. F: Quantification of colocalization of TGN46 with APP measured by ImageJ software. Statistics were analyzed using one-way ANOVA followed by Turkey’ posttest; n = 3 independent experiments. Mean ± SD. ∗*P* < 0.05, ∗∗*P* < 0.01, ∗∗∗*P* < 0.001.

In our study, we found that in DHCR24 knockdown neuronal cell lines, the amount of APP in early endosomes was slightly reduced, by analyzing the colocalization of fluorescence signals of APP and Rab5, a specific early endosome marker. However, in DHCR24 knockin neuronal cell lines, the amount of APP in early endosomes had not significantly changed (Fig. 4A, C). Moreover, we found that DHCR24 did not affect Rab5 protein expression, (Fig. 4D) an essential adaptor protein for endocytosis. However, the fluorescence puncta size of Rab5 per cell was smaller in DHCR24 knockin neuronal cell lines, compared to blank and vector cells. Contrarily, total early endosomes puncta area per cell was larger in DHCR24 knockdown neuronal cell lines (Fig. 4A, B). It has been reported that the numbers and morphology of early endosomes could suggest their functions (39); thus, increased areas of early endosomes indicated a dysfunctional early endosomes function in DHCR24 knockdown cells. Besides, by measured colocalization of fluorescence signals of APP with TGN46, a TGN-specific marker, we found that the amount of APP in TGN was increased in DHCR24 knockin neuronal cell lines compared to blank and vector cells while was reduced in DHCR24 knockdown neuronal cell lines, indicating decreased cellular cholesterol inhibited retrograde transport of APP from early endosome into TGN (Fig. 4E, F).

To sum up, our data suggest that cellular cholesterol deficiency could lead to disrupt a normal APP trafficking by impaired early endosome’s functions and hindered the transport of APP through early endosome to TGN.

### Cellular cholesterol deficiency by DHCR24 knockdown impaired APP trafficking regulated by cholesterol-associated proteins in HT22 neuronal cell lines

**Fig. 5:**
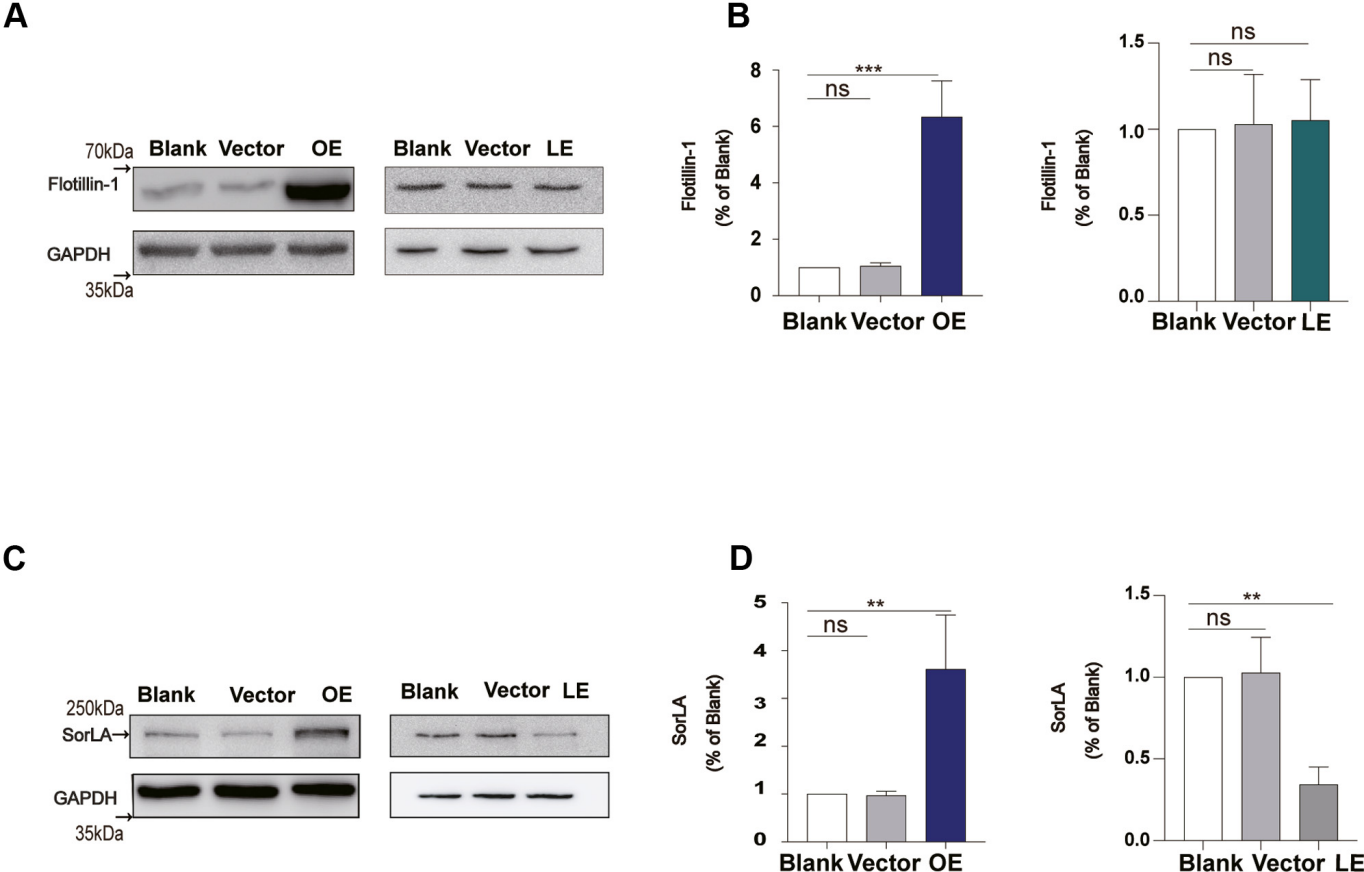
Cellular cholesterol deficiency by DHCR24 knockdown impaired APP trafficking regulated by cholesterol-associated proteins in HT22 neuronal cell lines. A: Representative western blots of Flotillin-1 from cell lysates from blank, vector, overexpressing (OE), and lower-expressing (LE) DHCR24 neuronal cell lines. B: Quantification of relative Flotillin-1/GAPDH ratio. C: Representative western blots of SorLA from cell lysates from blank, vector, OE, and LE DHCR24 neuronal cell lines. D: Quantification of relative SorLA/GAPDH ratio. Statistics were analyzed using one-way ANOVA followed by Turkey’ posttest; n = 3 independent experiments. Mean ± SD. ∗∗*P* < 0.01, ∗∗∗*P* < 0.001.

Cholesterol as an important component of membrane, extensively participates in vesicles trafficking. Moreover, several cholesterol-associated proteins have been found to play an important role in APP vesicles trafficking. To examine the effect of cholesterol on membrane-dependent endosomal recycling pathway for APP, we found the expressions of vesicles trafficking–associated proteins Flotillin-1 and sortilin-related receptor 1 (SORL1/SorLA) were altered in HT22 neuronal cell lines. Flotillin-1, as a critical constitutive protein of lipid rafts, played an essential role in vesicles trafficking, including APP intracellular trafficking (40, 41). In our study, consistent with elevated cellular cholesterol, we found that Flotillin-1 protein level was elevated in neuronal cell lines. Of note, although we did not observe any changes in Flotillin-1 protein level in DHCR24 knockdown neuronal cell lines (Fig. 5A, B), the reduced amount of APP localized in early endosomes could be a clue suggested cellular cholesterol loss may impair Flotillin-1–related membrane trafficking function.

SorLA is a sorting receptor involved in retrograding APP from early endosome into TGN (42, 43, 44). Similarly, we also found that DHCR24 could affect SorLA expression in neuronal cells. The expression of SorLA protein was significantly downregulated in DHCR24 knockdown neuronal cells, whereas was upregulated in DHCR24 knockin neuronal cell (Fig. 5C, D), suggesting enhancement of SorLA function in increased cholesterol condition. Interestingly, it has been shown that the expression of Flotillin-1 and SorLA was reduced in AD patients (45, 46, 47). Moreover, accumulating data confirm that depletion of SorLA significantly impairs the endosomal recycling pathway for APP, leading to the disruption in endosomal trafficking and increased Aβ deposits (44, 48). Therefore, our findings demonstrated that cellular cholesterol deficiency disrupted endosomal recycling pathway for APP by interfering APP trafficking-associated membrane protein function.

## DISCUSSION

Although the majority of the cell culture studies suggest that elevated cholesterol level increases Aβ production, a consensus has not yet been reached (6, 7, 8, 9). In our study, in hippocampal HT22 neuronal cell models of cellular cholesterol deficiency induced by DHCR24 knockdown, we found that deficiency of cellular cholesterol obviously increased intracellular and extracellular Aβ40/42 generation, conversely, in hippocampal HT22 neuronal cell models of increased cellular cholesterol level mediated by DHCR24 knockin, elevated cellular cholesterol markedly reduced the production of intracellular and extracellular Aβ40/42 (Fig. 2). In the meantime, in C8D1a astrocytic cells, we also observed that deficiency of cellular cholesterol induced by DHCR24 knockdown markedly promoted APP processing and increased intracellular and extracellular Aβ40/42 production. Elevation of cellular cholesterol level mediated by DHCR24 knockin reverses the increase of intracellular and extracellular Aβ40/42 generation in C8D1a astrocytic cells (Fig. 2). Most notably, our previous and other studies used human neuronal cell line called SH-SY5Y and have also made a similar conclusion of DHCR24’s effects on cholesterol and Aβ metabolism (11, 28). Additionally, it has been reported that upregulating PCSK9 expression in SH-SY5Y cell line inhibited neuronal cholesterol uptake and then increased Aβ neuronal toxicity (49). To sum up, the above results supported that cellular cholesterol deficiency induced Aβ generation or Aβ toxicity.

However, our outcomes and conclusion contradict the majority of previous work. This apparent contradiction prompted us to explore the role of cholesterol in Aβ generation in previous experimental systems. Firstly, similar to the majority of previous work, in the present study, we established neuronal HT22 and astrocytic C8D1A cell models with APP overexpression (Fig. 3). Surprisingly, we found that APP overexpression in both neuronal/ astrocytic cell lines lead to the decreased gene expression of main cholesterol synthetases, including *Srebp2*, *Hmgcr*, *Dhcr24*, *Dhcr7*, and *Sqle*, resulting in drastically decrease of cellular cholesterol level (Fig. 3). Similarly, numerous studies have shown that APP and its proteolytic fragments, such as APP intracellular domain, C99, Aβ40, and Aβ42, can markedly inhibit cellular cholesterol biosynthesis or impair cholesterol trafficking, leading to disruption of cellular cholesterol homeostasis (24, 25, 50). Apparently, APP or mutant APP knockin cell models represent a cell model of cellular cholesterol deficiency induced by APP overexpression and its proteolytic fragments. Secondly, previous studies have showed that overexpression of exogenous APP in APP knockin cell models might change the distribution of APP and other membrane-anchored proteins, which could alter its normal physiologic processing as well as the processing of other proteins (26, 51). Thirdly, in our experiment, in neuronal and astrocytic cells with APP overexpression, we showed that APP overexpression obviously increased APP processing and β-cleavage, suggesting increase of Aβ production (Fig. 3). Similarly, evidences revealed that overexpression of exogenous APP in APP knockin cell models obviously affect cellular APP processing and Aβ generation (26, 51). Because “addition” of exogenous APP into these membrane lipid-raft domains by means of overexpression may increase β-cleavage in a cholesterol-dependent manner, and the cleavage of overexpressing APP cells themselves may have occurred as a result of excess APP. So, we speculate that APP overexpression could affect the outcomes derived from the previous experimental systems. Overall, the APP overexpression of cell models in the previous experimental systems could not only disrupt cellular cholesterol homeostasis and affect structure and function of cell models but also promote Aβ production, which could affect the outcomes and conclusion (26, 51). Consequently, we suppose that APP knockin cell models maybe is not a suitable model for the study of the role of cholesterol in Aβ metabolism.

Noteworthily, compared with the previous cell models with APP overexpression, we found that the new cell models have three different features: (1) cellular APP expression level was not altered in the new cell models; (2) cellular cholesterol homeostasis also was not changed or disrupted in the new cell models before DHCR24 knockdown or knockin; and (3) cellular cholesterol levels could be effectively modulated by DHCR24 knockin or knockdown in the new cell models, which is consistent with our previous studies (28, 29, 30). In short, in present experiment, the new neuronal and astrocytic cell models could closely resemble physiological and pathological situations. Accordingly, we suppose that the new neuronal and astrocytic cell models could be a more suitable model in AD-cholesterol research. Based on previous data and our study, we concluded that there are obvious differences between the APP knockin cell models and our new cell models. Because of the differences between the new cell model and the APP knockin cell models, it gives rise to the controversial results and conclusion. Therefore, one rational explanation for the discrepancy between our outcomes and the previous results from the APP knockin cell models very likely to be attributed to the two different cell models. Taking together, we believe that the results and conclusion derived from APP knockin models could have to again be evaluated.

It was reported that treating astrocytes with proinflammatory cytokine or Aβ42 oligomers can increase APP and BACE-1 expressions in astrocytes and then made astrocytes secret more Aβ40 (19, 52). In addition, it was reported that astrocytes expressed APP and its related lyase proteins, indicating astrocytes equip with the machine to produce Aβ. Moreover, intracellular Aβ deposits were found in astrocytes (22, 23). Herein, we found that the generation of APP-derived β-CTFs, as well as intracellular/ secreted Aβ40 and 42 was increased in lower-expressing DHCR24 astrocytic cell lines. Hence, our outcomes and previous studies all confirmed that astrocytes could produce Aβ, and astrocyte could be a potential source of misfolded Aβ in the pathogenesis of AD.

Mechanistically, the molecular control of the initiation of APP processing and Aβ generation in endolysosomal dysfunction is poorly understood in AD (34, 35). Moreover, it was wildly acknowledged that APP in plasma membrane was highly sensitive to cellular cholesterol levels, and altered cellular cholesterol can affect APP endocytosis (53, 54). In our study, we found that cellular cholesterol depletion obviously altered the numbers of early endosomes which could be a sign of “cargo” traffic jam in early endosomes, suggesting the initial changes of AD pathogenesis (Fig. 4). Furthermore, in early-stage AD, intraneuronal accumulation of abnormally enlarged endosomes is common, indicating the endosomal recycling pathway for APP is disrupted, which is tightly related to Aβ generation (55). On the contrary, we found elevated cellular cholesterol level by DHCR24 knockin also could influence morphology of early endosomes, further evidenced that cholesterol involved in endosomes trafficking (Fig. 4). Besides, we showed that cellular cholesterol deficiency markedly decreased the expression of SorLA protein, indicating the impairment of SorLA function. Intriguingly, accumulating data validate that depletion of SorLA significantly impairs the endosomal recycling pathway for APP and lead to altered localization of APP in early endosomes, a site of APP β-cleavage, which contributes to β-amyloid generation and tau pathology (44, 48, 56). Taking together, our outcomes suggest that cellular cholesterol loss by DHCR24 knockdown leads to Aβ production by mediating endosomal recycling pathway for APP.

There is growing interest in the potential role of cholesterol in the pathogenesis of AD. Very importantly, genetic evidences from the inherited AD animals and patients, such as APOE4 allele, obviously supports that cellular cholesterol deficiency is associated with Aβ production and accumulation (4). Consistent with our present findings, increasing data reveal that cellular cholesterol deficiency could be involved in Aβ generation, tau hyperphosphorylation, apoptosis, synaptic injuries, inflammation, and autophagy (3, 4, 5, 57, 58, 59)). Taken together, our findings provide strong evidence that cellular cholesterol loss leads to the increase of Aβ generation which contributes to AD pathogenesis. Very importantly, because of the fact that increased cellular cholesterol mediated by overexpressing DHCR24 could reduce Aβ production and other AD-related pathological impairments, we believe that DHCR24 knockin could increase brain cellular cholesterol level as the potential treatment for AD (22).

## CONCLUSION

**Fig. 6:**
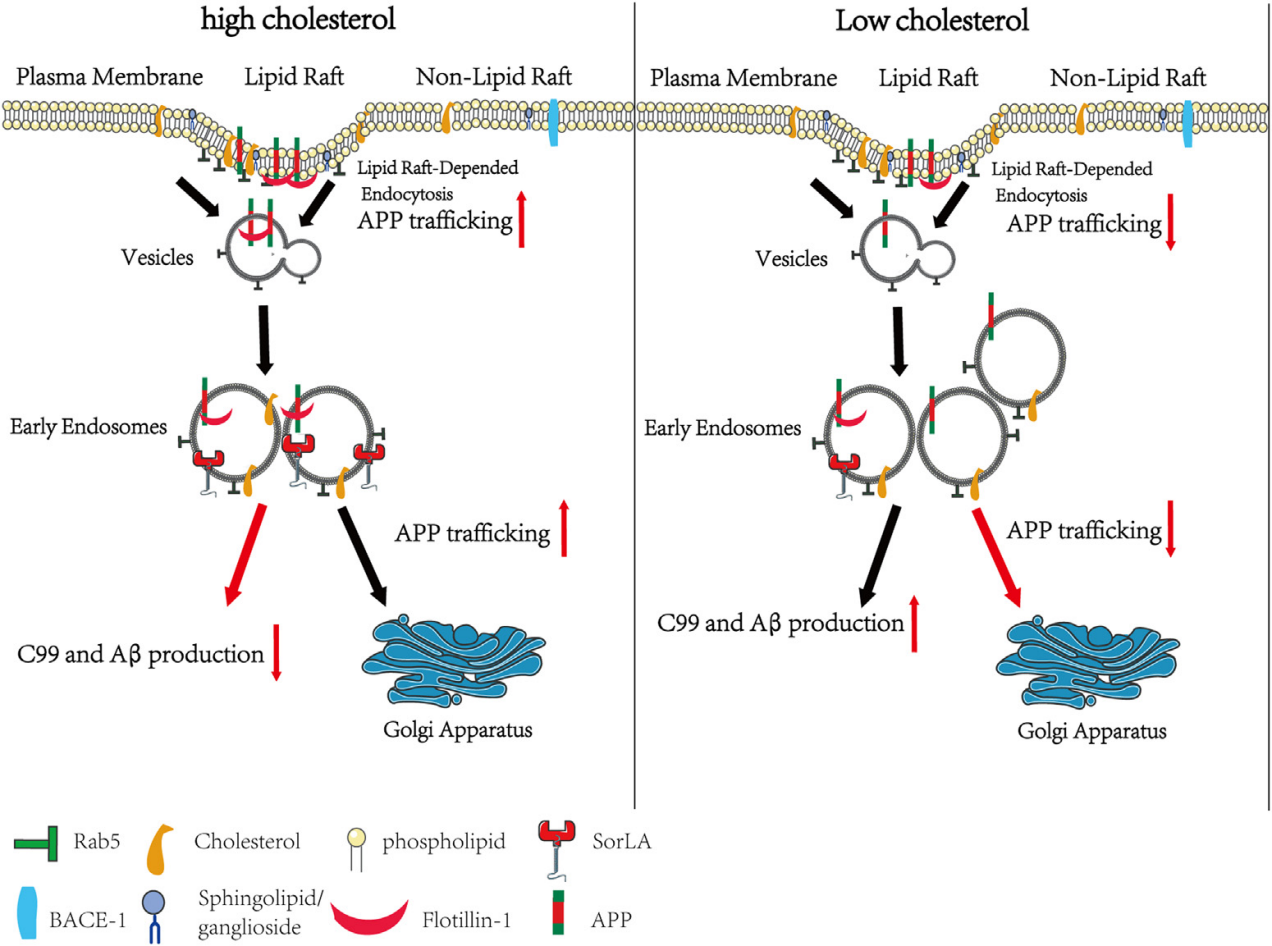
Schematic illustrating cellular cholesterol change APP intracellular localization in endocytic pathway. Physically, APP in plasma membrane is clustered by Flotillin-1 into cholesterol-coated pits. With the help of specific adaptor proteins like Rab5, APP was subsequently delivered into the early endosomes. And then partial APP in early endosomes was retrograde to TGN by SorLA. In cholesterol loss condition, the function of membrane-associated protein, like Flotillin-1 and SorLA, could be impaired, leading to defective vesicles trafficking. Moreover, the dysfunctional early endosomes could disrupt APP trafficking, leading to increased Aβ generation. By contrast, upregulating cellular cholesterol could elevate SorLA protein expression, which helped APP transport from early endosomes to TGN, resulting a reduced Aβ generation.

In summary, in new neuronal and astrocytic model systems mediated by DHCR24, we conclude that the deficiency of cellular cholesterol clearly plays an influential role in promoting Aβ production, at least partly by mediating cholesterol-dependent endosomal recycling pathway for APP (Fig. 6). Besides, we also confirmed that astrocytes could be an important source of Aβ production in the pathogenesis of AD. Based on our study and the previous data, we suppose that the conclusion derived from the APP knockin models will need to be re-evaluated.

## Data availability

All data supporting the conclusions of this article are available from the corresponding author on reasonable request.

## Supplemental data

This article contains supplemental data.

## Conflict of interest

The authors have no competing interests.
