# Supplementary material for: Cellular cholesterol loss by DHCR24 knockdown leads to Aβ production by changing APP intracellular localization
Source: J Lipid Res. 2023 Apr 1;64(5):100367. doi: 10.1016/j.jlr.2023.100367 (PMC10173783; doi:10.1016/j.jlr.2023.100367)
Supplement: Supplemental data [file mmc1.docx]

**Organized original pictures for figure1 in the text (from S-Figure 1-1 to S-Figure 1-2)**


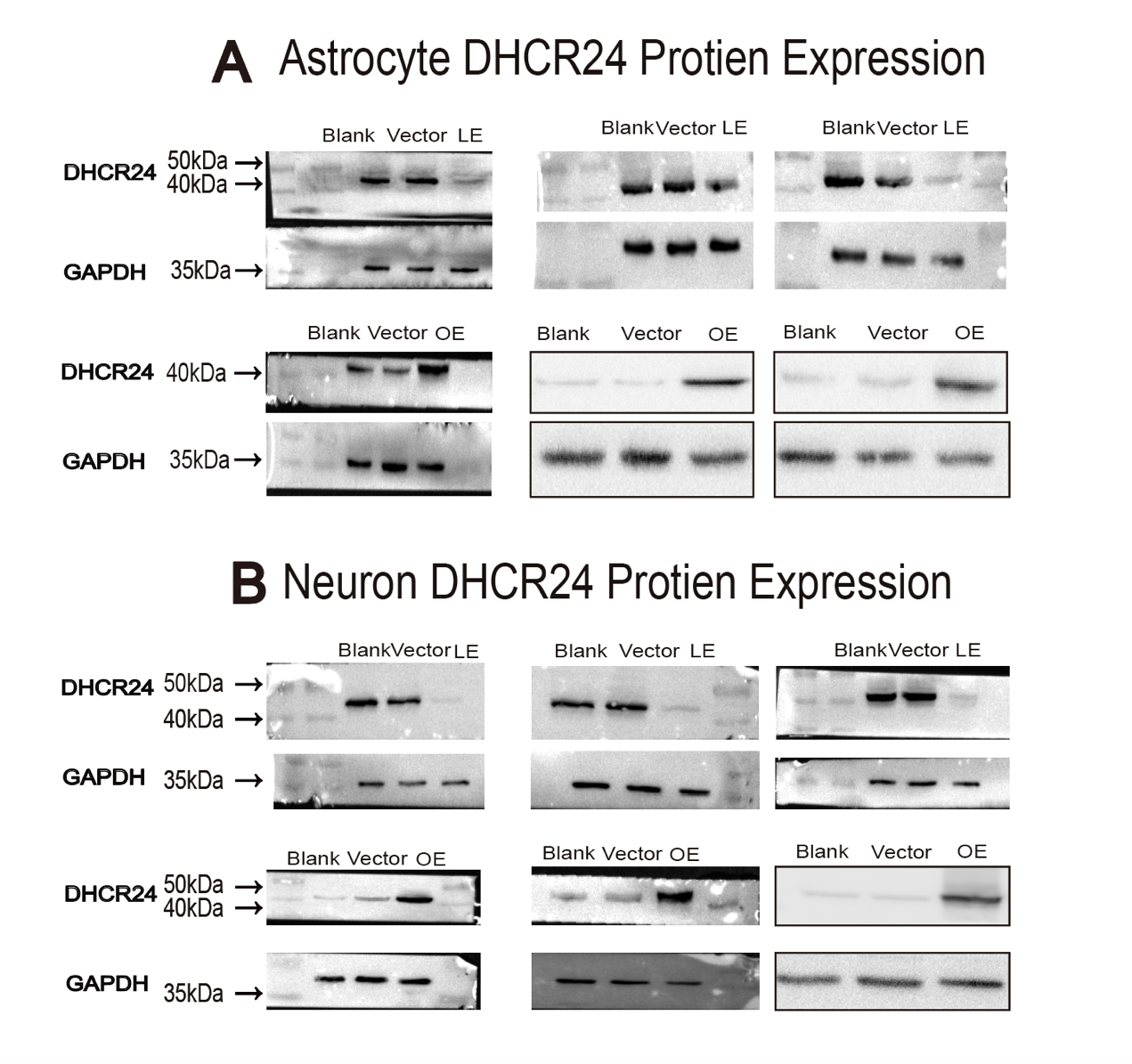


S-Figure 1-1 Organized original cropped images of immunoblots of DHCR24 and GAPDH of Blank, Vector, over-expressing (OE) and lower-expressing (LE) DHCR24 astrocytic and neuronal cell lines.


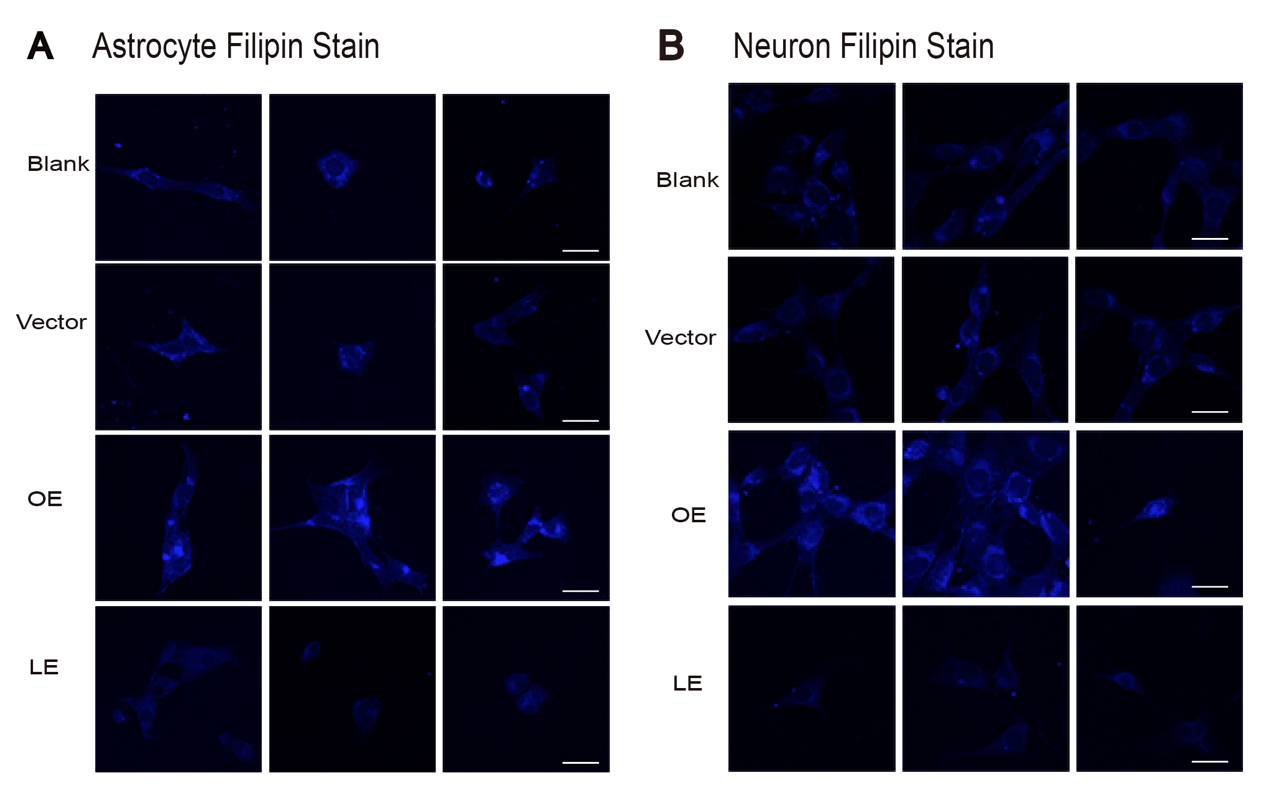
S-Figure 1-2 Organized original immunofluorescence images of in Blank, Vector, over-expressing (OE) and lower-expressing (LE) DHCR24 astrocytic and neuronal cell lines, which were stained by Filipin. Scale bar:20um.


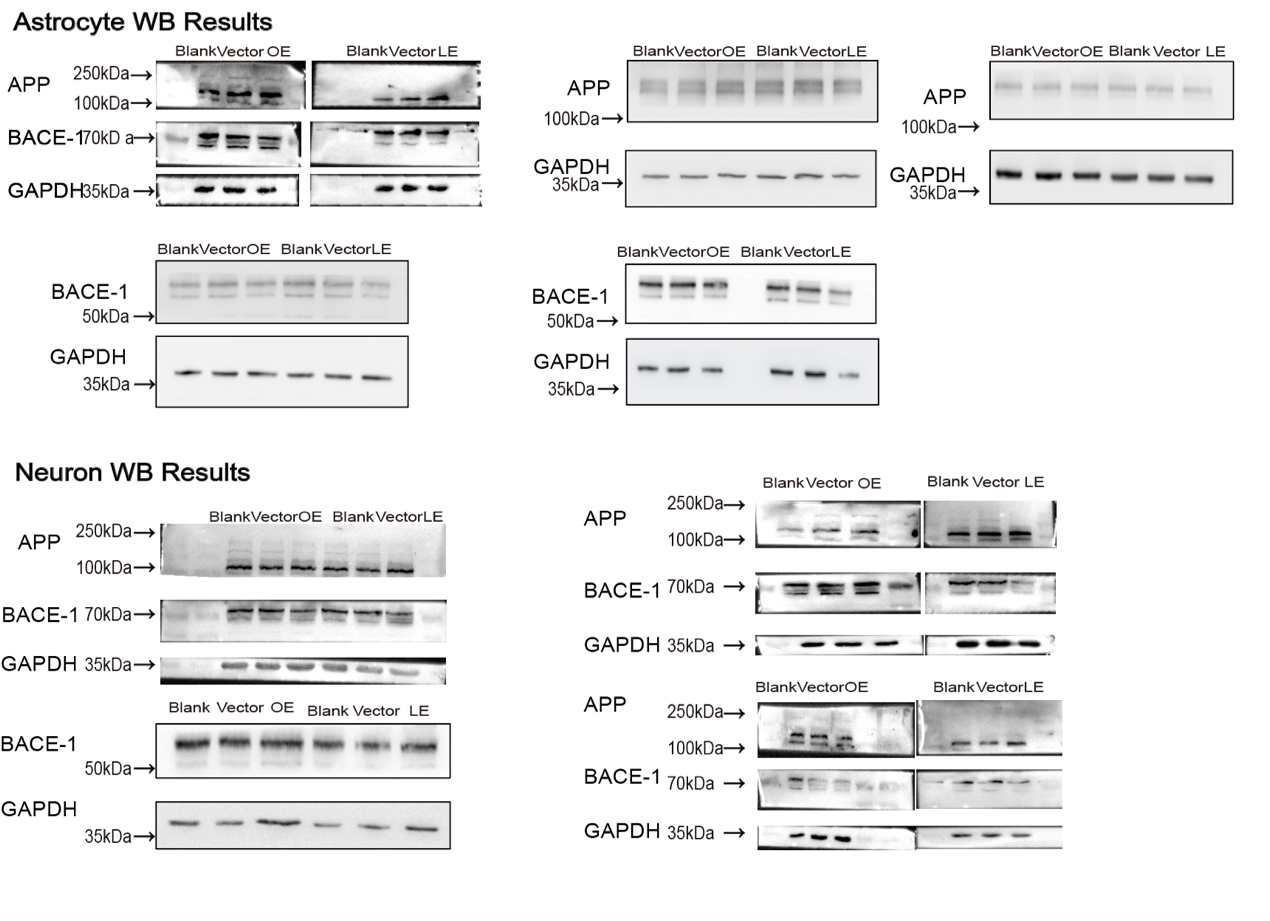
**Organized original pictures for figure2 in the text (from S-Figure 2-1 to S-Figure 2-2)**

S-Figure 2-1 Organized original cropped images of immunoblots of APP, BACE-1 and GAPDH of Blank, Vector, over-expressing (OE) and lower-expressing (LE) DHCR24 astrocytic and neuronal cell lines.

**
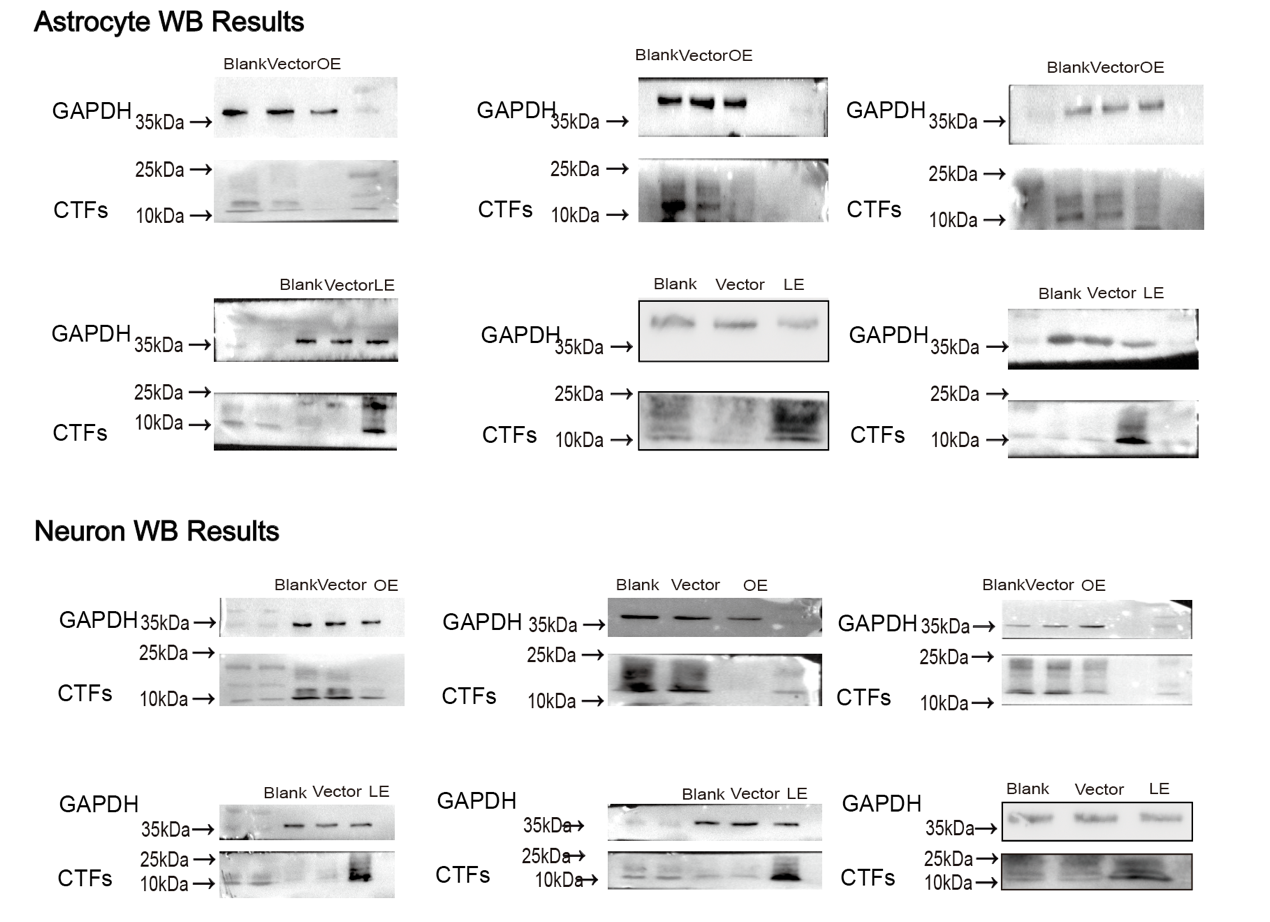
**

S-Figure 2-2 Organized original cropped images of immunoblots of CTFs (including C99 and C83) and GAPDH of Blank, Vector, over-expressing (OE) and lower-expressing (LE) DHCR24 astrocytic and neuronal cell lines.

**
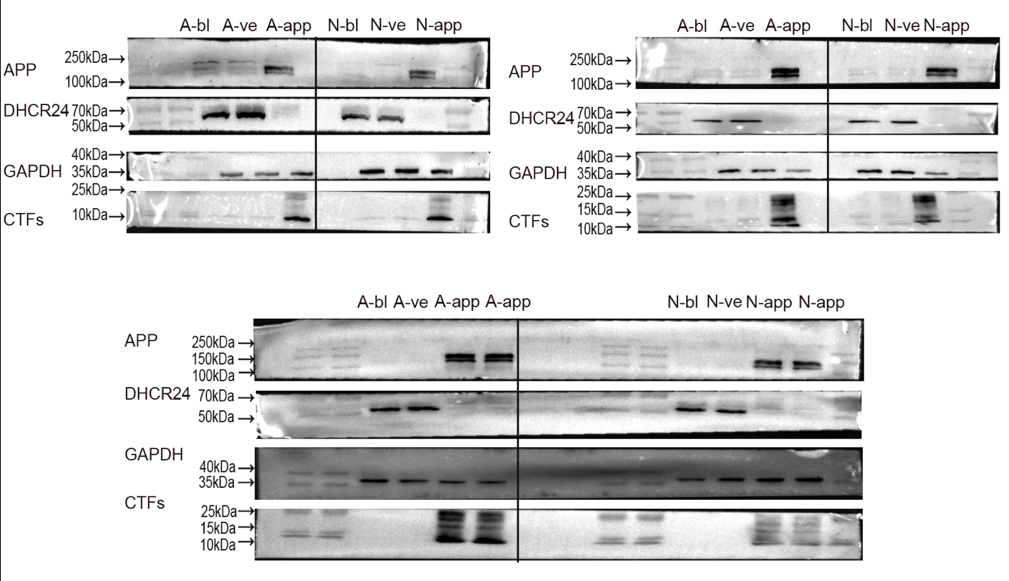
Organized original pictures for figure3 in the text (from S-Figure3-1 to S-Figure 3-2 )**


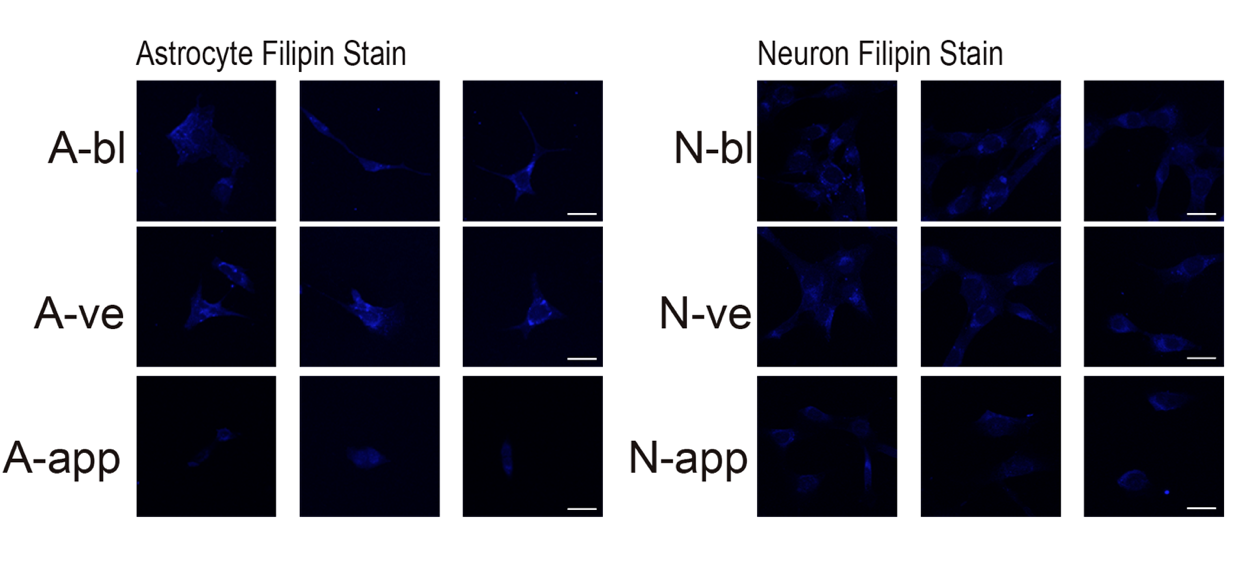
S-Figure 3-1 Organized original cropped images of immunoblots of APP, DHCR24, CTFs (including C99 and C83) and GAPDH of Blank, Vector, APP-knock in astrocytic and neuronal cell lines. Astrocytic blank group was abbreviated with A-bl, astrocytic vector group was abbreviated with A-ve, astrocytic APP-knock in group was abbreviated with A-app. Neuronal blank group was abbreviated with N-bl, neuronal vector group was abbreviated with N-ve, neuronal APP-knock in group was abbreviated with N-app.

S-Figure 3-2 Organized original immunofluorescence images of in Blank, Vector, APP knock-in astrocytic and neuronal cell lines, which were stained by Filipin. Scale bar:20um. Astrocytic blank group was abbreviated with A-bl, astrocytic vector group was abbreviated with A-ve, astrocytic APP-knock in group was abbreviated with A-app. Neuronal blank group was abbreviated with N-bl, neuronal vector group was abbreviated with N-ve, neuronal APP-knock in group was abbreviated with N-app. Note: N-bl group share same Filipin immunofluorescence images with neuron blank group in S-Figure 1-2 showed above, since these data are analyzed for Filipin stain immunofluorescence mean intensity in blank neuron.


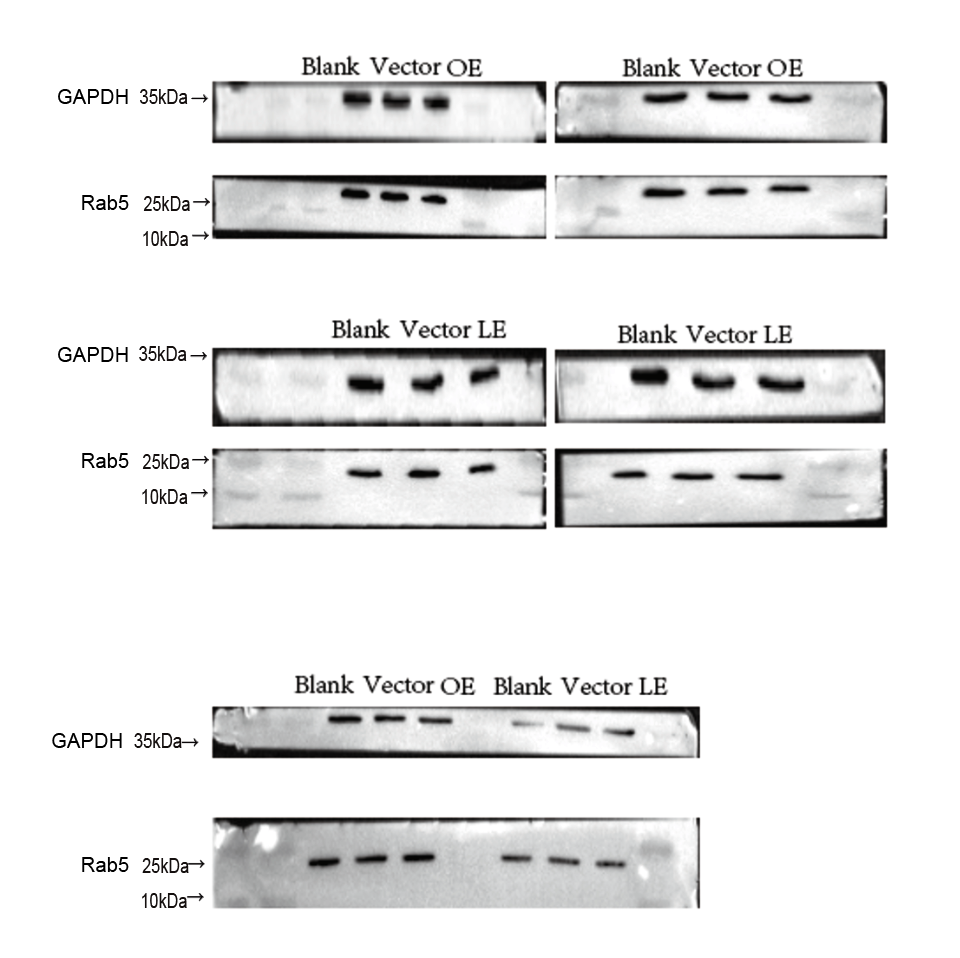
 **Organized original pictures for figure4 in the text**

S-Figure 4 Organized cropped images of immunoblots of Rab5 and GAPDH of Blank, Vector, over-expressing (OE) and lower-expressing (LE) DHCR24 neuronal cell lines.


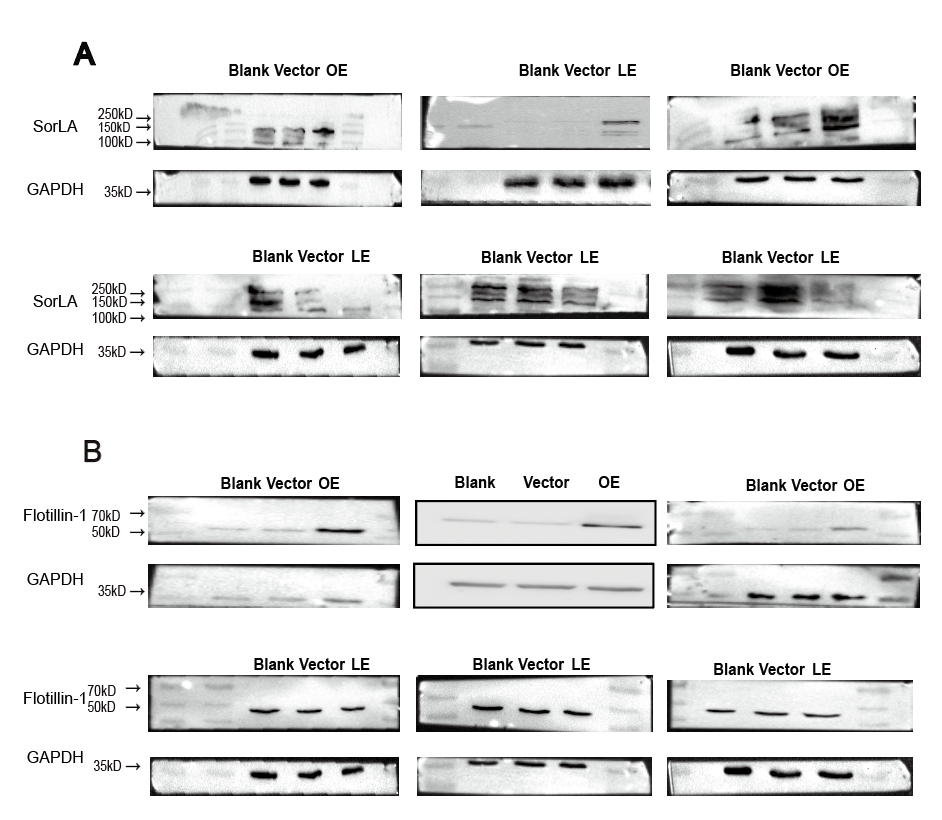
**Organized original pictures for figure5 in the text**

S-Figure 5 Organized original cropped images of immunoblots. (A) Representative western blots of SorLA and GAPDH of Blank, Vector, over-expressing (OE) and lower-expressing (LE) DHCR24 neuronal cell lines. (B) Representative western blots of Flotillin-1 and GAPDH of Blank, Vector, over-expressing (OE) and lower-expressing (LE) DHCR24 neuronal cell lines. Note: in neuronal group, images of immunoblots of Rab5 (26kDa), GAPDH (37kDa), Flotillin-1(47kDa) and SorLA (250kDa) were cropped from a same membrane, thus (A) and (B), and S-Figure4 and S-Figure5 shared the same GAPDH immunoblot images.
